# The Offprint of an Abnormal Pre-Parotidectomy Electrodiagnostic Finding in a Normally Functioning Facial Nerve: Correlation with Intraoperative Findings, Histology and Postoperative Facial Nerve Function †

**DOI:** 10.3390/jcm11010248

**Published:** 2022-01-04

**Authors:** Verena Katheder, Matti Sievert, Sarina Katrin Müller, Vivian Thimsen, Antoniu-Oreste Gostian, Matthias Balk, Robin Rupp, Heinrich Iro, Konstantinos Mantsopoulos

**Affiliations:** Department of Otolaryngology, Head and Neck Surgery, Friedrich-Alexander Universität Erlangen-Nuremberg (FAU), Waldstrasse 1, 91054 Erlangen, Germany; verena.katheder@fau.de (V.K.); Matti.sievert@uk-erlangen.de (M.S.); sarina.mueller@uk-erlangen.de (S.K.M.); vivian.thimsen@uk-erlangen.de (V.T.); antoniu-oreste.gostian@uk-erlangen.de (A.-O.G.); Matthias.Balk@uk-erlangen.de (M.B.); robin.rupp@uk-erlangen.de (R.R.); heinrich.iro@uk-erlangen.de (H.I.)

**Keywords:** facial nerve, electroneurography, electromyography, parotidectomy, infiltration, tumor, facial paralysis

## Abstract

The aim of this study was to search for associations between an electrodiagnostically abnormal but clinically normal facial nerve before parotidectomy and the intraoperative findings, as well as the postoperative facial nerve function. The records of all patients treated for parotid tumors between 2002 and 2021 with a preoperative House–Brackmann score of grade I but an abnormal electrophysiologic finding were studied retrospectively. A total of 285 patients were included in this study, and 222 patients had a benign lesion (77.9%), whereas 63 cases had a malignant tumor (22.1%). Electroneurographic facial nerve involvement was associated with nerve displacement in 185 cases (64.9%) and infiltration in 17 cases (6%). In 83 cases (29.1%), no tumor–nerve interface could be detected intraoperatively. An electroneurographic signal was absent despite supramaximal stimulation in 6/17 cases with nerve infiltration and in 17/268 cases without nerve infiltration (*p* < 0.001). The electrophysiologic involvement of a normal facial nerve is not pathognomonic for a malignancy (22%), but it presents a rather rare (~6%) sign of a “true” nerve infiltration and could also appear in tumors without any contact with the facial nerve (~29%). Of our cases, two thirds of those with an anatomic nerve preservation and facial palsy had already directly and postoperatively recovered to a major extent in the midterm.

## 1. Introduction

It is a common surgical experience that the vast majority of preoperatively intact facial nerves can be dissected macroscopically free from a parotid gland tumor [1,2]. The information that facial nerve preservation during parotid surgery is possible in almost all patients with preoperatively normal facial nerve function is important for alleviating a patient’s fear of potential postoperative complete facial paralysis [3]. On the other hand, paralysis of the facial nerve due to a parotid gland lesion is a strong clinical sign of a tumorous infiltration of the facial nerve in the presence of malignancy. However, the absence of this rather rare condition does not clinically exclude a malignancy, as it is more or less common knowledge that the majority of malignant tumors of the parotid gland are present as unsuspicious lesions, according to clinical imaging findings [4,5]. Facial nerve paralysis due to tumor infiltration occurs only in 12–15% of these cases [2]. Interestingly, facial nerve palsy does not always involve a malignant tumor. In very rare cases, inflammation caused by a benign tumor (e.g., cystadenolymphoma) can lead to temporary dysfunction of the facial nerve [6]. Tumorous infiltration of the facial nerve due to a parotid malignancy is not always associated with abnormal pre-therapeutic facial nerve function. Detection of a subtle functional loss in facial nerve electrodiagnostics due to a parotid gland tumor could provide some proof of malignance [4,5,7,8]. Invasion of the facial nerve upgrades the T category to T4a (“moderately advanced local disease”) and the disease to stadium I [9] with a significant impact on prognosis. Concerning counseling and surgical planning, both surgeon and patient should be prepared in such cases for the potential intraoperative need for nerve resection and reconstruction [10]. The rationale behind the current study is the need to investigate the clinical value (“offprint“) of a diagnostic test, which has been long established in our department. A review of the relevant literature revealed only two older studies with a limited number of patients dealing with the value of preoperative facial nerve electrodiagnostics in the management of parotid gland tumors [5,6,7,8]. In the present study, a significantly larger number of cases with electroneurographic involvement of a clinically normal facial nerve (House–Brackmann [11] grade I) due to a parotid gland tumor were investigated for a potential relationship between the electrophysiologic parameters, histology and postoperative facial nerve function, as well as the intraoperative findings (exact topographic relationship between the nerve and the tumorous lesion).

## 2. Materials and Methods

This study was conducted at an academic tertiary referral center specializing in salivary gland diseases (Department of Otorhinolaryngology, Head and Neck Surgery, University of Erlangen–Nuremberg, Erlangen, Germany). The records of all patients treated for tumors of the parotid gland between 2002 and 2021 with preoperatively clinically normal facial nerve function (House–Brackmann [11] grade I) but with an abnormal finding in the routine preoperative facial nerve electrodiagnostic examination were studied retrospectively. Patients with insufficient data or previous surgery in the ipsilateral parotid gland were excluded from further analysis.

**Figure 1:**
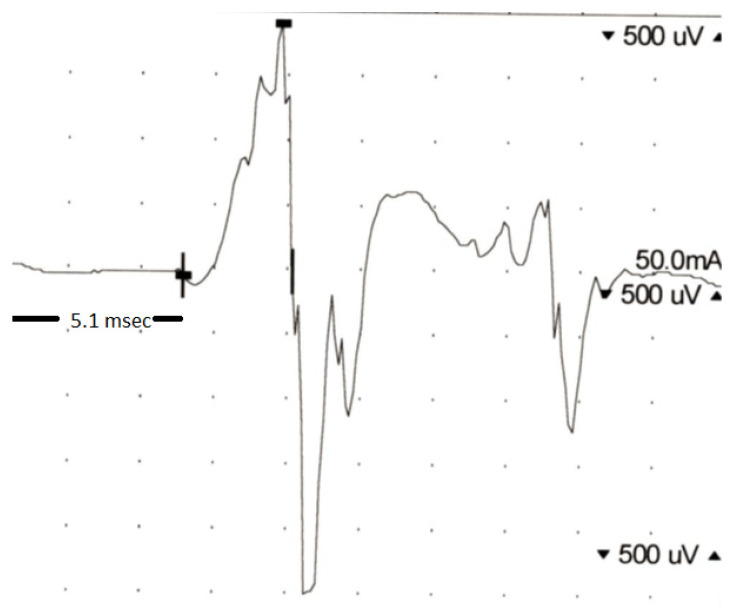
Latency of 5.1 ms (>4.0 ms) in electroneurography (nerve conduction test) of the facial nerve.

**Figure 2:**
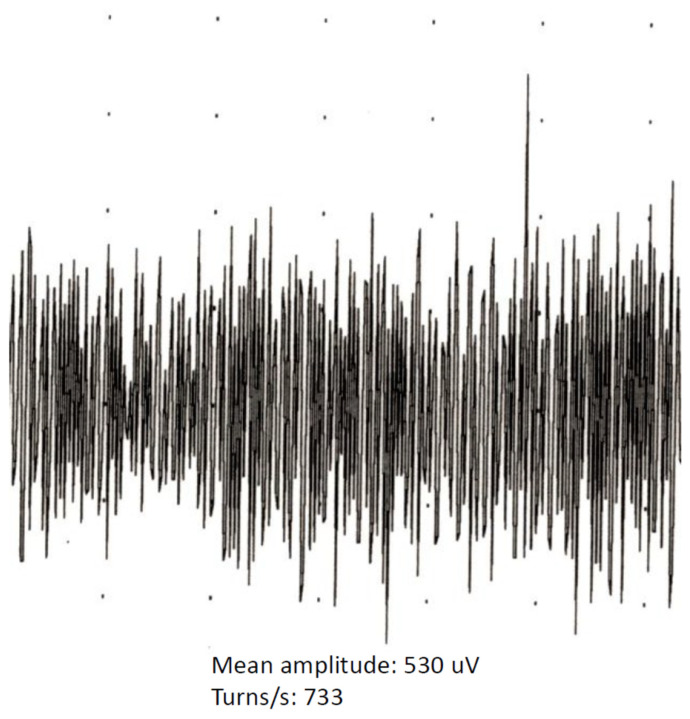
Electromyography of the facial nerve with turns amplitude analysis.

Electroneurography (ENoG) as well as needle electromyography (nEMG) are standard investigation tools used routinely before parotidectomy in our department. ENoG analyzes the evoked compound muscle action potential (CMAP) of the caudal mimic muscles after transcutaneous stimulation of the main trunk of the facial nerve to a maximum of 40 mA at its exit from the stylomastoid foramen with a bipolar stimulator, as recommended in the relevant literature [7,8,9,10,11,12]. The CMAP is recorded using a bipolar (concentric) pair of needle electrodes placed in the nasolabial fold [7,8,9,10,11,12,13]. In accordance with the relevant literature [5,14,15,16], a pathologic finding in ENoG was defined as a latency >4.0 ms in the nerve conduction test (Figure 1). In the qualitative analysis of the EMG, we sought possible signs of muscle degeneration (e.g., positive sharp waves or fibrillation potentials). Quantitative analysis of the EMG signal was performed by means of turns amplitude analysis (ΤAA). A “turn” is defined as a change in the polarity of the EMG signal when the amplitude difference between changes is greater than 100 mV [17,18,19]. The TAA parameters considered were the mean amplitude, turns per second as well as the turns-to-amplitude ratio (Figure 2). All preoperative EMG examinations were performed with the Nicolet EDX EMG/NCS/EP/IOM System with Natus Elite Software (Natus, Inc., Middleton, MA, USA). In all cases, intraoperative neuromonitoring was performed to identify, protect or dissect (if necessary) the main trunk or the branches of the facial nerve (Medtronic or Inomed). Intraoperative neuromonitoring consisted of one stimulation probe and two electrodes for conducting the action potentials of the orbicularis oculi and orbicularis oris muscles. At the beginning, stimulation was carried out with a maximum current of 4–5 mA. As soon as a branch of the facial nerve was identified by positive stimulation CMAPs, the stimulating current was reduced to 1 mA [20]. The function of the facial nerve was assessed using the House–Brackmann grading system [11] immediately postoperatively (day of surgery) and at the last patient presentation (mean follow-up: 16 months, SD: ±6.9).

The aim of our study was to investigate the clinical offprint of a pathologic preoperative electroneurographic finding of the facial nerve and to search for potential associations with the dignity and the size (maximal diameter) of the parotid lesion, the intraoperative topographic relationship of the tumor to the facial nerve (e.g., no nerve–tumor contact, nerve displacement or nerve infiltration) and facial nerve function in the direct and long-term postoperative phase. Statistical analysis was performed using the *t*-test as well as the x^2^ test with 95% confidence intervals (CIs). SPSS version 21 software for Windows (SPSS, Inc., Chicago, IL, USA) was used for the analysis. A *p*-value of <0.05 was considered statistically significant. The Institutional Review Board (IRB) of the University Hospital of Erlangen approved this study.

## 3. Results

In a total of 4105 cases, two hundred eighty-five (285) patients (6.9%) were detected with preoperatively clinically normal facial nerve function (House–Brackmann [11] grade I) but with an abnormal finding in the routine preoperative facial nerve electrodiagnostic examination and were included in the study (190 men, 95 women; male/female ratio: 2:1). The mean age was 63.1 years (range: 27–92 years). A total of 222 patients had a benign lesion (77.9%), whereas 63 cases had a malignant parotid gland tumor (22.1%). Histology of the parotid gland tumors is shown in Table 1 and Table 2. Out of the 63 malignant cases, 24 were primary malignant tumors of the parotid gland (38.1%), and the remaining 39 cases had lesions of a non-salivary gland origin (61.9%). From the 24 malignant tumors of a salivary gland origin, 10 (41.7%) were “low-grade”, 4 were intermediate (16.6%) and 10 were “high-grade” (41.7%). Only in two cases were there tumors with the characteristic perineural invasion (adenoid cystic carcinomas). In total, preoperative electroneurographic involvement of the facial nerve was intraoperatively attributed to a displacement of the nerve (with preservation of nerve continuity) in 185 cases (64.9%) and to a nerve infiltration in 17 cases (6%). In 83 cases (29.1%), no interface (contact) between the tumorous lesion and the facial nerve branches could be detected intraoperatively. An electroneurographic signal was absent despite supramaximal stimulation of the facial nerve with 40 mA in 6/17 cases with infiltration of the facial nerve and in 17/268 cases without nerve infiltration (*p* < 0.001). Concerning the pattern of intraoperative nerve–tumor interference, infiltration of the nerve was significantly more frequent in the group of malignant tumors (*p* < 0.001, Table 3). The single case with infiltration of the nerve by a benign tumor (Table 1) showed a lesion growing at the stylomastoid foramen in the entire circumference of the facial nerve. The mean size of the maximal diameter of the lesions of the study sample was 31.2 mm (±12.9 mm). The size of the lesions with infiltration of the facial nerve branches was larger (33.2 ± 13.6 mm) in comparison with the tumors with simple displacement of the facial nerve (31.7 ± 13.2 mm), which in turn were larger than the tumors without contact to the nerve (29.3 ± 12.9 mm). In this context, no statistical significance was achieved (*p* = 0.311). The analysis of the TAA data is presented in Table 4. The mean follow-up was 16 months (SD: ±6.9). Data on the postoperative facial nerve function are presented in Table 5. Comparative analysis of the cases with displacement of the nerve and without any contact with the nerve showed a statistically significant difference in favor of the second group directly postoperatively (*p* = 0.002). However, this difference was eliminated by the patients’ last presentation (*p* = 0.288).

We monitored the facial nerve function of all 83 of these cases at regularly scheduled follow-up examinations at 6 weeks, 3 months, 6 months and at least 12 months after surgery or until facial palsy had completely recovered. Out of these 83 cases, 54 recovered completely in the first 12 months. In this subgroup of 54 patients, 10 cases (all with slight palsies) had recovered completely by discharge (3rd postoperative day), as well as another 36 cases between the 3rd day and 3 months after surgery. The remaining 8 cases recovered completely in the time interval between 3 and 12 months so that at the follow-up examination in about 3 months, the difference between the displaced and non-displaced facial nerves had become insignificant.

## 4. Discussion

Guntinas-Lichius et al. considered ENoG as well as EMG to be indispensable diagnostic tools in the detailed assessment of preoperative facial function in patients with head and neck neoplasms [21]. In our department, electrodiagnostics of the facial nerve is a standard investigation tool used routinely before the surgical management of all lesions in the direct vicinity of the facial nerve (e.g., parotid gland tumors, cholesteatomas and acoustic neuromas). The rationale behind the standardized performance of facial nerve electrodiagnostics before parotidectomy lies in the conviction that nerve involvement due to a parotid tumor could be attributed, among other factors, to an entrapment neuropathy. As in most of the common nerve compression syndromes (e.g., carpal tunnel syndrome, ulnar nerve lesions at the elbow and common peroneal nerve lesions at the fibular head), electroneurography (nerve conduction study) is one of the first-choice diagnostic modalities [22,23]. Adopting the same diagnostic concept in our cases could reasonably complement the preoperative workup of our patients. Additionally, pre-parotidectomy electrophysiologic examination of the facial nerve has been traditionally considered a means for indirect identification of tumor dignity, as almost exclusively malignant lesions are known to invade the facial nerve. Furthermore, these diagnostic modalities can raise the suspicion of infiltration of the facial nerve due to a confirmed malignancy and contribute to the optimization of patient counseling and treatment planning. Third, these diagnostic tools enable the detection of a preexisting subtle (subclinical) functional loss of the facial nerve, which could be relevant in medicolegal issues.

The incidence of malignant tumors in our study sample (22%) was almost the same as the incidence of malignancy in the general population with parotid tumors (~20%). In contrast to our former long-established belief, this observation points to the fact that an abnormal finding in facial nerve electrodiagnostics is by no means pathognomonic for a malignant tumor. Our data showed that only a total of 6% of our cases had malignant infiltration of the facial nerve intraoperatively. Almost half (49.2%) of the malignant cases caused a displacement of the nerve without any signs of invading it. This finding corresponds to the high incidence of low-grade subtypes (41.7%) with a far less aggressive behavior toward the nerve. In our salivary gland registry, squamous cell carcinomas in elderly patients tend to form one of the largest groups of malignant tumours in the parotid gland with an increasing tendency over the last decades (potentially an indirect sign of the ageing population of oncologic patients). Squamous cell carcinomas in the parotid gland are considered to be rather metastatic cutaneous manifestations of this entity in the intraparotideal lymph nodes (21 cases in the group of the 39 cases with a non-salivary gland origin). The high rate of this entity in a study examining the electroneurographic tumour-associated facial nerve subclinical involvement matches the known aggressive (infiltrative) behaviour of this tumour towards the facial nerve.

It is established knowledge that the vast majority of patients with malignant lesions of the parotid gland present with unspecific and not alarming symptoms [24,25]. Facial nerve paralysis is present in less than one sixth of these cases [2]. Therefore, detecting a malignancy in this group of patients only by means of clinical examination and imaging requires experience and expertise in the interpretation of imaging findings, and it is often quite challenging for the clinician [24,26]. Guntinas-Lichius et al. stated that electrodiagnostics is more sensitive than the clinical examination itself in detecting infiltration of the facial nerve due to a parotid gland lesion [21,27]. Our data sustained this statement, as electrophysiologic studies in the preoperative phase “uncovered” another 8 malignant tumors infiltrating the facial nerve with otherwise inconspicuous clinical features.

Routine electromyographic clinical practice is mostly qualitative and tends to rely on a rather subjective interpretation of the signals. In general, EMG could allow distinguishing between a neurogenic and a myogenic lesion in many cases, as the latter is characterized by an increase in the mean amplitude and a reduction in turns/second. A further sign of muscle denervation is provided though the typical spontaneous muscle activity in the needle EMG, characterized by positive sharp waves and fibrillation potentials [28]. However, qualitative analysis of the EMG of the mimic muscles is complicated by the similarity as well as the overlap of findings between pathologic and normal conditions [17]. In this context, TAA represents an effort to quantify electromyographic findings [29]. However, experience in applying this method to the mimic muscles is very limited and rather disappointing due to its low sensitivity [17], and it is only scarcely reported in the relevant literature [17,30,31]. In our study, a comparison of cases with and without tumorous infiltration of the facial nerve revealed a significantly higher value of turns/second in the second group. Evaluation of this parameter in a preoperative EMG could offer a reliable basis for preoperative identification of cases with infiltration of the facial nerve as well as optimization of treatment planning and patient counseling.

Remarkably, almost 30% of our study patients had lesions without any contact with the nerve, and as such they did not show any intraoperative correlation for the abnormal electrophysiologic finding. In trying to explain this apparent discrepancy, we thought that this finding could be explained either by a preexisting non-tumorous nerve irritation (e.g., post-infectious or inflammatory) or an intraparenchymal indirect transfer of tension by a voluminous tumor distant from the facial nerve. Reasonably, the rigid, hardly expandable parotid parenchyma with multiple fibrous septa offers the ideal conditions for such an assumption. However, this last explanation alone is not sufficient for the high number of lesions smaller than 20 mm (36/83, 43.4%) in this subgroup of cases. In any case, as almost one third of the study cases did not show any contact with the facial nerve, the abnormal electrodiagnostic finding of a clinically normal facial nerve should not lead to the preoperative abandonment of a minimal invasive procedure per se (e.g., extracapsular dissection) if palpation and imaging are able to sustain this treatment strategy.

**Figure 3:**
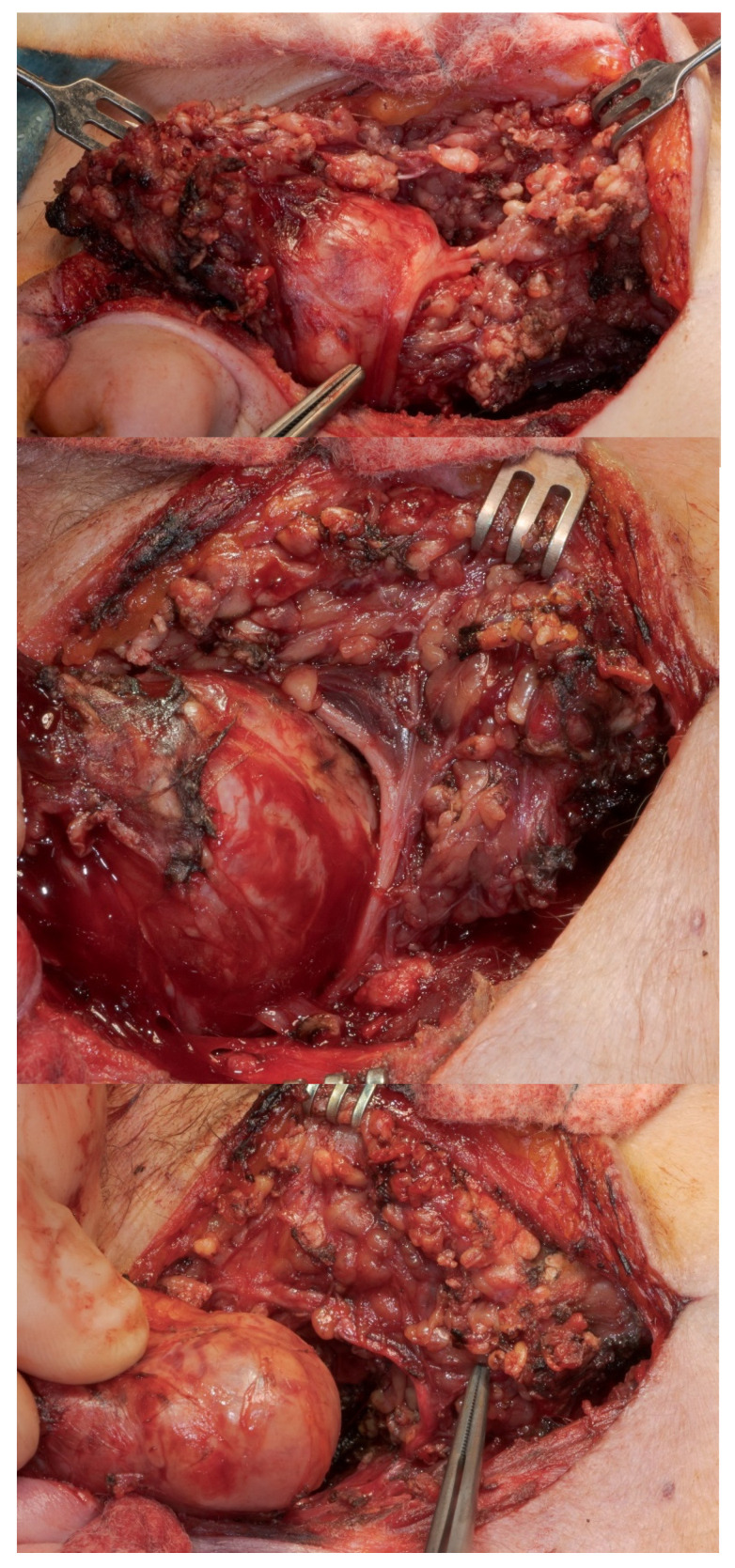
Benign tumor of the right parotid gland (histology: pleomorphic adenoma), with massive adhesions and major displacement of the main trunk and bifurcation of the facial nerve.

Concerning the postoperative functional outcome, a total of 100/285 patients (35.1%) suffered from facial palsy (irrespective of severity) in the direct postoperative phase. If we exclude the 17 cases with infiltration of the facial nerve (and the consequent unavoidable nerve resection and loss of mimic function), this incidence in the remaining 268 cases would be as high as 30.9%. At the time of last presentation, the facial palsy rate fell to 10.8% (29/268 cases), with the majority of the cases showing only a slight (House–Brackmann grade II, 22 cases, 8.2%) or moderate paralysis (House–Brackmann grade III, 4 cases, 1.5%). This means that two thirds of the cases with postoperative dysfunction of an anatomically intact facial nerve experienced a complete restitution of their palsy, and 97.3% of all study patients had, under consideration of the demanding intraoperative condition (nerve displacement and compression), an acceptable midterm functional outcome throughout (House–Brackmann grade I and II). It seems that the edema and hyperemia of the nerve already seen intraoperatively, due to the burdensome surgical manipulation for detaching the tumor from the nerve (Figure 3), led to temporary palsy in these cases, which probably resolved completely when the swelling decreased. When matching this possible explanation in 15 of the cases with major displacement of the main trunk, we observed that facial nerve function was normal on the first postoperative day but deteriorated progressively in the next few days. In all of these cases, function of the facial nerve returned to normal in the short term (up to 6 weeks). In the 17 cases with nerve infiltration, resection of the main trunk was necessary in 6 cases (6/17, 35.3%). In the remaining cases, the tumorous lesions most frequently infiltrated the marginal mandibular or buccal branches of the facial nerve.

A compromise we had to make was associated with the need to describe the postoperative outcome of facial nerve function in a homogeneous manner. The House–Brackmann grading system refers to lesions involving the main trunk (e.g., Bell’s palsy or postoperative outcome after surgery for acoustic neuromas) and measures the function of the facial nerve in a global manner. Unfortunately, we could not find any other way to homogeneously describe the postoperative facial nerve function irrespective of the site of involvement of the nerve (main trunk vs. branch). Furthermore, in the cases without nerve infiltration, the postoperative facial nerve outcome is undoubtedly related not only to the preoperatively existing abnormalities but also to a number of intraoperative parameters (e.g., surgical manipulation by different surgeons, varying expertise and experience and different surgical philosophies). This confusing factor could not be eliminated because of the large number of different surgeons operating on these cases. A further potential limitation of the current study is associated with the selective potential recording from the nasolabial fold. Kim et al. stated that ENoG of the nasolabial fold has more prognostic value in the outcome of Bell’s palsy than recording of the orbicularis oculi [13]. Guntinas-Lichius et al. accepted that the nasolabial fold is the most reliable recording site for ENoG [7]. The nasolabial folds are maintained by the orbicularis oris, zygomaticus major, zygomaticus minor, levator labii superioris and levator labii superioris alaeque nasi muscles. These muscles are mostly innervated by the zygomatic, buccal and marginal mandibular branches. That means that the majority of the facial nerve branches (except for the temporal branch) can be examined though recording in the nasolabial fold. Interestingly, in no case were we confronted with isolated involvement of the peripheral part of the temporal branch of the facial nerve (which selectively innervates the ipsilateral frontal muscle). For this reason, and in order to simplify the examination and decrease discomfort for the patient, we decided to place paired electrodes only in the nasolabial fold. However, we have to accept that testing of both the orbicularis oculi and the nasolabial fold would have been theoretically more sensitive in the investigation of our cases.

## 5. Conclusions

When studying 286 cases, we observed that the EMG involvement of a clinically normal facial nerve was by no means pathognomonic for a malignancy (22%), was a rather rare (~6%) sign of a “true” nerve infiltration and could remarkably also appear in tumors without any contact with the facial nerve (~29%). In the latter cases, an abnormal finding in the facial nerve electrodiagnostics could possibly be attributed to a preexisting non-tumorous nerve irritation (e.g., post-infectious or inflammatory) or an intraparenchymal indirect transfer of pressure or tension to the facial nerve. A careful evaluation of the quantitative EMG parameters could be promising for reliably detecting facial nerve infiltration preoperatively. Two thirds of our cases with facial palsy occurring directly postoperatively already recovered to a major extent in the mid-term.

## Figures and Tables

**Table 1:** Histology of the benign tumors of our study sample.

Histology	Number of Cases	%
Cystadenolymphoma (“Warthin’s tumor”)	108	48.6
Pleomorphic adenoma	72	32.5
Basal cell adenoma	10	4.5
Oncocytic adenoma	7	3.2
Chronic sialadenitis	6	2.7
Lymphoepithelial cyst	4	1.8
Parotid cyst	4	1.8
Lymphadenitis	3	1.4
Oncocytic hyperplasia	3	1.4
Myoepithelioma	2	0.9
Abscess	1	0.5
Monomorphic adenoma	1	0.5
Schwannoma	1	0.5
Total	222	100

**Table 2:** Histology of the malignant tumors of our study sample.

Histology	Number of Cases	%
“Low-grade” mucoepidermoid carcinoma	4	6.3
“Low-grade” acinic cell carcinoma	4	6.3
Salivary duct carcinoma (ex-pleomorphic adenoma)	4	6.3
Salivary duct carcinoma (“de novo”)	2	3.2
Adenoid cystic carcinoma	2	3.2
Adenocarcinoma	2	3.2
Dedifferentiated acinic cell carcinoma	1	1.6
Dedifferentiated mucoepidermoid carcinoma	1	1.6
“Low-grade” basal cell adenocarcinoma	1	1.6
“Low-grade” intraductal carcinoma (“intercalated cell” type)	1	1.6
Undifferentiated large cell carcinoma	1	1.6
Epithelial myoepithelial carcinoma	1	1.6
Lymphoma	12	19
Malignant melanoma	3	4.8
Merkel cell carcinoma	2	3.2
Parotid gland metastasis from breast cancer	1	1.6
Squamous cell carcinoma	21	33.3
Total	63	100

**Table 3:** Correlation of the histology of the tumors of the study sample with the intraoperatively detected pattern of anatomic nerve involvement in the tumor.

	Histology		
	Benign Tumors	Malignant Tumors	Total
Pattern of nerve involvement			
Displacement	154	31	185
Infiltration	1	16	17
No tumor–nerve interface	67	16	83
Total	222	63	285

**Table 4:** Comparative analysis of the patient groups with and without infiltration of the facial nerve on the basis of data derived from the turns amplitude analysis. bold: statistically significant.

	No Facial Nerve Infiltration	Facial Nerve Infiltration	*p* Value
Mean number of turns/second (±SD)	871.9 ± 369.6	723.8 ± 304.7	**0.04**
Mean amplitude (±SD)	3684.9 ± 1685.2	3420.6 ± 2007.5	0.274
Mean turns/amplitude ratio	0.26 ± 0.10	0.25 ± 0.09	0.4

**Table 5:** Comparative analysis of the patient groups with displacement of the facial nerve and without any contact to the nerve concerning postoperative facial nerve function.

	Direct Postoperative Phase	Last Presentation	Direct Postoperative Phase	Last Presentation
House–Brackmann	Displacement		No Tumor Nerve Contact	
I	113	160	72	79
II	47	19	9	3
III	15	3	0	1
IV	5	0	1	0
V	4	2	1	0
VI	1	1	0	0

## Data Availability

The data presented in this study are available on request from the corresponding author.

## References

[1] Vander Poorten V., Bradley P.J., Takes R.P., Rinaldo A., Woolgar J.A., Ferlito A. (2012). Diagnosis and management of parotid carcinoma with a special focus on recent advances in molecular biology. Head Neck.

[2] Pedersen D., Overgaard J., Sogaard H., Elbrond O., Overgaard M. (1992). Malignant parotid tumors in 110 consecutive patients: Treatment results and prognosis. Laryngoscope.

[3] Guntinas-Lichius O., Klussmann J.P., Schroeder U., Quante G., Jungehuelsing M., Stennert E. (2004). Primary Parotid Malignoma Surgery in Patients with Normal Preoperative Facial Nerve Function: Outcome and Long-Term Postoperative Facial Nerve Function. Laryngoscope.

[4] Wiertel-Krawczuk A., Huber J., Wojtysiak M., Golusiński W., Pieńkowski P., Golusinski P. (2015). Correlations between the clinical, histological and neurophysiological examinations in patients before and after parotid gland tumor surgery: Verification of facial nerve transmission. Eur. Arch. Oto-Rhino-Laryngol..

[5] Aimoni C., Lombardi L., Gastaldo E., Stacchini M., Pastore A. (2003). Preoperative and Postoperative Electroneurographic Facial Nerve Monitoring in Patients with Parotid Tumors. Arch. Otolaryngol. Head Neck Surg..

[6] Mantsopoulos K., Psychogios G., Agaimy A., Künzel J., Zenk J., Iro H., Bohr C. (2013). Inflamed benign tumors of the parotid gland: Diagnostic pitfalls from a potentially misleading entity. Head Neck.

[7] Guntinas-Lichius O., Volk G.F., Olsen K.D., Mäkitie A.A., Silver C.E., Zafereo M.E., Rinaldo A., Randolph G.W., Simo R., Shaha A.R. (2020). Facial nerve electrodiagnostics for patients with facial palsy: A clinical practice guideline. Eur. Arch. Oto-Rhino-Laryngol..

[8] Bendet E., Talmi Y.P., Kronenberg J. (1998). Preoperative electroneurography (ENoG) in parotid surgery: Assessment of facial nerve outcome and involvement by tumor—A preliminary study. Head Neck.

[9] (2017). AJCC Cancer Staging Manual.

[10] Volk G.F., Pantel M., Guntinas-Lichius O. (2010). Modern concepts in facial nerve reconstruction. Head Face Med..

[11] House J.W., Brackmann D.E. (1985). Facial Nerve Grading System. Otolaryngol. Neck Surg..

[12] Ayani Y., Haginomori S.-I., Wada S.-I., Nakano H., Inaka Y., Ozaki A., Ichihara T., Inui T., Kawata R. (2021). Optimal current intensity for supramaximal stimulation during electroneurography for facial palsy. Auris Nasus Larynx.

[13] Zwaveling S., Steenvoorde P., Da Costa S.A. (2006). Treatment of Postparotidectomy Fistulae with Fibrin Glue. Acta Medica.

[14] Skevas A.T., Danielides V.G., Assimakopoulos D.A. (1990). The role of the facial nerve latency test in the prognosis of Bell’s palsy. Laryngoscope.

[15] de Medeiros J.L., Nobrega J.A., de Andrade L.A., Juliano Y. (1996). Facial nerve electroneurography. Variability in normal subjects. Arq. Neuropsiquiatr..

[16] Ayani Y., Haginomori S.-I., Wada S.-I., Nakano H., Hamada M., Ichihara T., Inui T., Inaka Y., Ozaki A., Kawata R. (2019). Latency shift in compound muscle action potentials during electroneurography in facial palsy. Eur. Arch. Oto-Rhino-Laryngol..

[17] Farrugia M., Kennett R. (2005). Turns amplitude analysis of the orbicularis oculi and oris muscles. Clin. Neurophysiol..

[18] Willison R.G. (1964). Analysis of electrical activity in healthy and dystrophic muscle in man. J. Neurol. Neurosurg. Psychiatry.

[19] Stålberg E., Nandedkar S.D., Sanders D.B., Falck B. (1996). Quantitative Motor Unit Potential Analysis. J. Clin. Neurophysiol..

[20] Bär B., Mantsopoulos K., Iro H. (2020). Paradigm shift in surgery for benign parotid tumors: 19 years of experience with almost 3000 cases. Laryngoscope.

[21] Guntinas–Lichius O. (2004). The facial nerve in the presence of a head and neck neoplasm: Assessment and outcome after surgical management. Curr. Opin. Otolaryngol. Head Neck Surg..

[22] Schulte-Mattler W.J., Grimm T. (2015). Common and not so common nerve entrapment syndromes: Diagnostics, clinical aspects and therapy. Nervenarzt.

[23] Assmus H., Martini A.-K. (2010). Nerve compression syndromes of the upper extremity. Z. Orthopädie Unf..

[24] Mantsopoulos K., Koch M., Iro H. (2017). Extracapsular dissection as sole therapy for small low-grade malignant tumors of the parotid gland. Laryngoscope.

[25] Mantsopoulos K., Mueller S., Goncalves M., Koch M., Iro H. (2019). Completion surgery after extracapsular dissection of low-grade parotid gland malignant tumors. Head Neck.

[26] Mantsopoulos K., Velegrakis S., Iro H. (2015). Unexpected Detection of Parotid Gland Malignancy during Primary Extracapsular Dissection. Otolaryngol. Neck Surg..

[27] Guntinas-Lichius O., Silver C.E., Thielker J., Bernal-Sprekelsen M., Bradford C.R., De Bree R., Kowalski L.P., Olsen K.D., Quer M., Rinaldo A. (2018). Management of the facial nerve in parotid cancer: Preservation or resection and reconstruction. Eur. Arch. Oto-Rhino-Laryngol..

[28] Stitik T.P., Foye P., Nadler S.F. (1999). Electromyography in craniomaxillofacial trauma. J. Cranio-Maxillofac. Trauma.

[29] Fuglsang-Frederiksen A. (2000). The utility of interference pattern analysis. Muscle Nerve.

[30] Karandreas N., Kararizou E., Papagianni A., Zambelis T., Kokotis P. (2010). Turns-amplitude analysis in normal and myopathic facial muscles. Muscle Nerve.

[31] Finsterer J. (2006). Differentiation between neurogenic and myogenic lesions of facial muscles by turn/amplitude analysis. Clin. Neurophysiol..

